# Cognitive Functions in Repeated Glioma Surgery

**DOI:** 10.3390/cancers12051077

**Published:** 2020-04-26

**Authors:** Gabriele Capo, Miran Skrap, Ilaria Guarracino, Miriam Isola, Claudio Battistella, Tamara Ius, Barbara Tomasino

**Affiliations:** 1 Department of Neurosurgery, Azienda Ospedaliero-Universitaria Santa Maria della Misericordia, 33100 Udine, Italy; 2 Scientific Institute, IRCCS E. Medea, San Vito al Tagliamento, 33078 Pordenone, Italy; 3 Department of Medical and Biological Sciences, Section of Statistics, University of Udine, 33100 Udine, Italy

**Keywords:** awake surgery, neurocognition, neuropsychological assessment, diffuse low surgery

## Abstract

Low-grade gliomas (LGG) are slow-growing brain tumors infiltrating the central nervous system which tend to recur, often with malignant degeneration after primary treatment. Re-operations are not always recommended due to an assumed higher risk of neurological and cognitive deficits. However, this assumption is relatively ungrounded due to a lack of extensive neuropsychological testing. We retrospectively examined a series of 40 patients with recurrent glioma in eloquent areas of the left hemisphere, who all completed comprehensive pre- (T3) and post-surgical (T4) neuropsychological assessments after a second surgery (4-month follow up). The lesions were most frequent in the left insular cortex and the inferior frontal gyrus. Among this series, in 17 patients the cognitive outcomes were compared before the first surgery (T1), 4 months after the first surgery (T2), and at T3 and T4. There was no significant difference either in the number of patients scoring within the normal range between T3 and T4, or in their level of performance. Further addressing the T1–T4 evolution, there was no significant difference in the number of patients scoring within the normal range. As to their level of performance, the only significant change was in phonological fluency. This longitudinal follow-up study showed that repeated glioma surgery is possible without major damage to cognitive functions in the short-term period (4 months) after surgery.

## 1. Introduction

Low-grade gliomas (LGG) are a heterogeneous group of primary brain tumors that often arise in young, otherwise healthy, patients, often located close to or actually infiltrating eloquent brain areas. In the case of these tumors, survival is associated with maximal extent of resection (EOR) [1,2,3,4,5,6,7,8,9], though this may affect neurological and cognitive functions, and consequently, patient quality of life (QoL). Management is then based on histology, molecular profile, tumor location, and patient characteristics. To combine maximal resection with a reduced risk of clinical and neuropsychological impairment, mapping with awake craniotomies and neuropsychological testing has become an almost mandatory stage in therapeutic management in supratentorial locations. We know that LGG tend to recur, both migrating along subcortical pathways and growing into the previous cavity, evolving towards a higher grade of malignancy in a high percentage of cases [9,10,11,12,13,14,15]. Only a few recent surgical studies [3,16,17,18] have provided evidence that repeated surgery improves the chances of overall survival (OS) and progression-free survival (PFS). Several clinicians are reluctant to re-operate on LGG patients, preferring new cycles of chemotherapy and/or radiotherapy, their major concern related to making further, serious damage to cognitive function in exchange for poor prognostic benefit. However, cortical reorganization of functional brain areas and the remodeling of neural circuitry may allow the re-operation of previously un-removable lesions, and help to preserve cognitive function in the presence of an evolving structural lesion [19,20,21].

Despite the amount of available data on this topic, the role and impact of re-resection in the management of recurrent glioma has not been clearly defined [3]. In this study, we sought to elucidate the impact of re-operation on the neurocognitive skills of patients affected by glioma recurrence.

### Aim of the Study

The aim of our study was to assess the neuropsychological performance in patients who undergo a recurrent surgery. In particular, we measured whether the impact of a second surgery on neuropsychological performance was significantly higher than the impact a first surgery might have. The literature on this topic is currently scarce.

We assessed the cognitive functioning of a consecutive series of 40 patients (mean age was 37 years, range 15−66 y) with recurrent glioma in eloquent areas [22,23,24] of the left hemisphere, treated by the senior author (M.S.) between 2010 and 2017, before (T3) and after (T4) second surgery. All patients were evaluated by two physicians (an attending neurosurgeon and a neuropsychologist) at least three times during the perioperative period of re-operation: one week before surgery, one week after surgery, and 4 months later. A subset of patients of this series (n = 17; mean age was 35 years, range 20–56 y), the control group, were assessed also before (T1) and after (T2) their first surgery. Patients had no comorbidities or other symptoms, with the exception of epilepsy in some cases. They underwent surgery the first time, then a repeat surgery on the recurrence of the disease years later. For all the patients, surgery was performed at our institution. All the 40 patients had T3–T4 neuropsychological data. Only 17 had complete T1–T4 neuropsychological data because the remaining underwent first surgery years before the introduction of extensive pre- and post-surgery neuropsychological testing.

All tumors were left-hemispheric, with histological confirmation of an infiltrative glioma at the time of initial surgery and showed progression in their MRI (magnetic resonance imaging) sequence, often with symptomatic worsening (seizures).

## 2. Results

**Figure 1:**
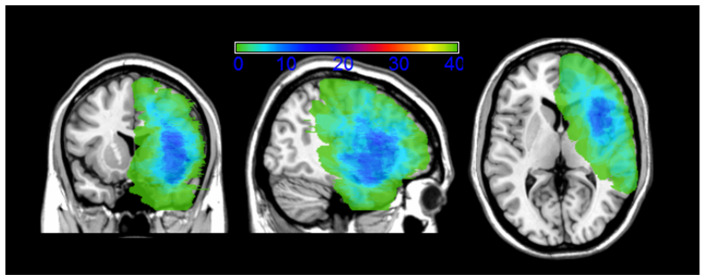
Overlap of all the 40 pre-recurrence (T3) patients’ lesions masks. Lesion masks include both the surgical cavity of the first surgery and the lesion regrowth. The number of overlapping lesions is illustrated by different colors coding increasing frequencies (as indicated in the bar code). As shown by the image, the epicenter of the overlap is located at the level of the frontotemporoinsular region. MR images are displayed in radiological convention.

The final study population was comprised of 40 patients. Mean (±SD) age was 37 (±11) years, mean (±SD) educational level or years of schooling was 12.7 ± 4 years, with 68% male and 32% female patients who were right-handed (92.1 ± 20.2 mean handedness). The subgroup of patients (N = 17) that were tested at four time points (T1–T4) had a mean age of 35 ± 8.78 and a mean years of schooling of 13.5 ± 3.6 years and were right-handed (94.4 ± 12.7 mean handedness). The baseline clinical, radiological, and histopathological characteristics of the patients are summarized in Table 1. All the tumors were located in the left hemisphere, and in all the cases the recurrence was local (see Figure 1). However, the time between the first and recurrent surgery was variable (mean months 49.2 ± 38.6). Previous treatments were antiepileptic treatments and MRI controls at 6/8 months post-surgery. fMRI data were routinely acquired, and results determined that the left hemisphere was the dominant one.

**Figure 2:**
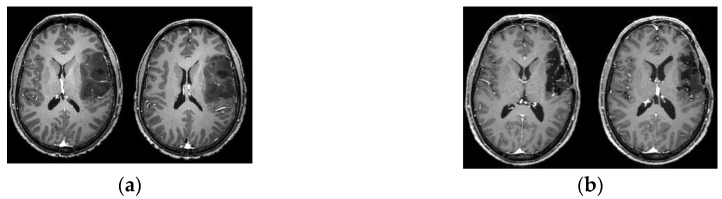
Axial view T1-weighted MRI. Upper image shows frontotemporoinsular glioma before first surgery. (**a**) Lower images show recurrence before second surgery. (**b**) Recurrent neoplastic tissue was located both along the walls of and within the previous-tumor cavity.

Eight gliomas were involved in the fronto-parietal lobe (somatosensory–motor cortex), 23 were in the paralimbic system (9 frontoinsular, 1 temporoinsular, 13 frontotemporoinsular), 5 involved the temporal lobe (operculum), 2 were in the frontal operculum, and 2 were in the temporo-parietal lobe. Figure 1 shows the lesion overlaps. Recurrent lesions were often located both along the walls of and within the previous-tumor cavity (see Figure 2 for a representative case).

According to the 2016 WHO classification of the brain tumors, based on morphology and molecular alterations, 15 tumors remained astrocytoma, and 2 were oligodendroglioma, while 16 patients had progressed to anaplastic status, and 7 had progressed to grade IV status at recurrence.

### 2.1. Neuropsychological Data

Table 2 shows the pre- and post-surgery number of patients within the normal range of performance for all the analyzed cognitive tasks. Some patients improved at T4, some worsened at T4, and the others remained unchanged, namely some patients were below the normal range at T3 and remained so at T4.

**Figure 3:**
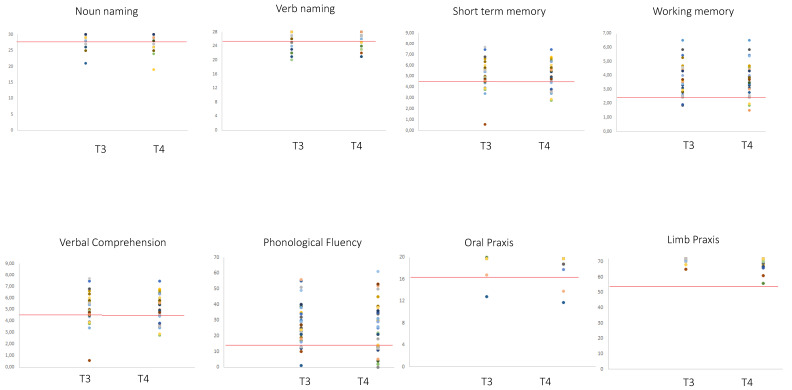
Performance of the 40 patients with recurrent glioma at T3 and T4 (pre- and post-recurrence surgery, respectively). Red lines indicate the cut-off, below which the patient results were pathological: verbal short-term memory (<4.26 is equal to an equivalent score of 0), working memory (<2.65 is equal to an equivalent score of 0), ideomotor limb apraxia (<53) [25] and oral apraxia (<16) [26], language comprehension (<26.25 is equal to an equivalent score of 0) [27], object naming and action naming (<28 and <26 respectively) [28], and verbal fluency (<16 is equal to an equivalent score of 0) [29]. Patients showing identical scores are represented by overlapping dots.

**Figure 4:**
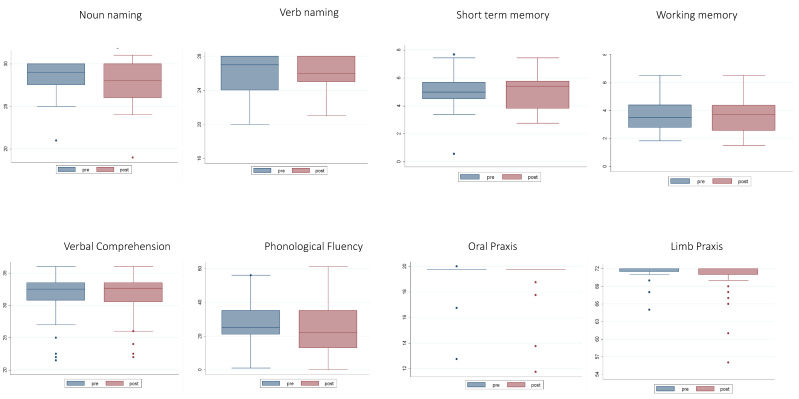
Mean performance of the 40 patients with recurrent glioma T3 and T4 (pre- and post-recurrence surgery, respectively). Bars represent the standard deviations.

The analyses of the number of patients exhibiting normal performance at T4 vs. T3 did not provide evidence of significant differences. The analyses of the level of performance (working memory and phonological fluency data were normally distributed; whereas comprehension, oral and ideomotor apraxia, short-term memory, noun and verb naming data were not normally distributed (see Table 3)), showed that except for the oral praxis, even remaining within the normal range, in which a minimal difference between a pre-surgery level of 19.5 ± 1.19 and a post-surgery level of 19.3 ± 1.5 was evidenced, there was no significant difference between T3 vs. T4 performance for any of the other tasks (Table 4 and Figure 3 and Figure 4).

**Figure 5:**
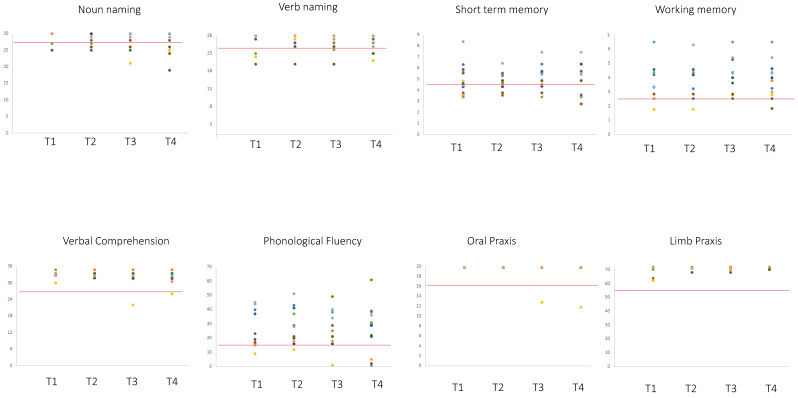
Performance of the 17 patients with recurrent glioma at T1 and T2 (pre- and post-first surgery, respectively) and at T3 and T4 (pre- and post-recurrence surgery, respectively). Red lines indicate the cut-off, below which the patient results are pathological (<4.26 is equal to an equivalent score of 0) [30,31], working memory (<2.65 is equal to an equivalent score of 0) [30], ideomotor limb apraxia (<53) [25] and oral apraxia (<16) [26], language comprehension (<26.25 is equal to an equivalent score of 0) [27], object naming and action naming (<28 and <26 respectively) [28] and verbal fluency (<16 is equal to an equivalent score of 0) [29]. Patients showing identical scores are represented by overlapping dots.

**Figure 6:**
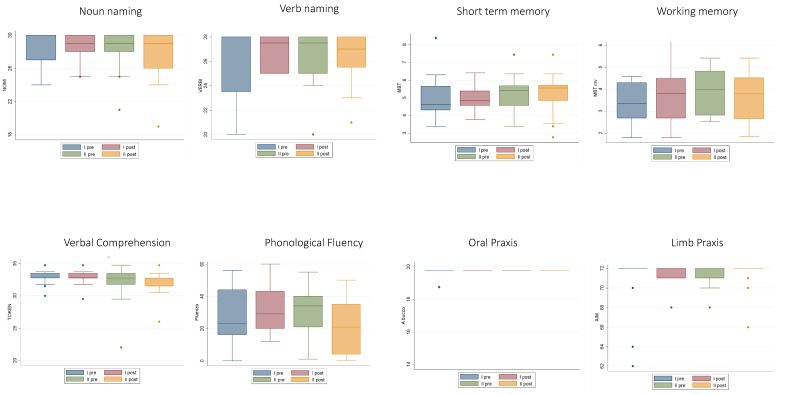
Mean performance of the 17 patients with recurrent glioma T1 and T2 (pre- and post-first surgery, respectively) and T3 and T4 (pre- and post-recurrence surgery, respectively). Bars represent the standard deviations.

We further addressed the neuropsychological pattern for the subgroup (N = 17) of patients who were tested pre- and post- first and second surgery (T1–T4). The analyses of the number of patients exhibiting normal performance in the T1–T4 measures did not evidence significant differences (Table 5). The analyses of the level of performance, repeated measures ANOVA (Table 6) showed that among all the tests, only phonological fluency showed a significant change in results between T4 and T1, between T4 and T2, and between T4 and T3 (see Figure 5 and Figure 6).

### 2.2. Radiotherapy

No effect was observed as a result of conventional radiotherapy performed after the recurrent surgery (see Table 7).

### 2.3. MRI Structural Data Analysis

Figure 1 shows the overlap of the patients’ lesion masks (lesion masks include both the surgical cavity of the first surgery and the lesion regrowth). This overlay of the patients’ lesion masks shows that the lesions involved eloquent areas. The maximum overlap of the pre-surgical lesion masks, occurring in at least 25% of the patients with recurrence surgery (N = 40), involved the inferior frontal gyrus (pars opercularis). We performed a Voxel Lesion Symptom Mapping (VLSM) analysis trying to correlate lesion location and neuropsychological performance. No significant voxels survived the multiple comparisons corrected threshold.

### 2.4. Intraoperative Findings in Recurrence Surgery

In first surgery, all the patients were operated under the awake condition. The same approach was used in recurrent surgery, since the indications and justifications were the same as for the first surgery, given that the same area was involved in the recurrent resection.

At recurrent surgery, from a technical/surgical point of view there was the appearance of the scar left by the first surgery, while resection was actually facilitated by the presence of recurring pathological tissue in the surgical cavity, which was well defined and easily distinguishable from the healthy tissue. As far as the infiltrative component was concerned, the methodological testing remained the same.

All lesions were on the dominant left-hand side, where language-related areas were the most frequent regions exposed by the craniotomy. Therefore, the intra-surgery changes most often involved language-related functions: seven patients exhibited a moderate decrease in language performance, with a reduction in naming and fluency. Three patients showed transitory fluctuations in language performance, two patients were subject to generalized seizures, and one exhibited extreme drowsiness at the end of the procedure.

### 2.5. Extent of Resection

The EOR was computed in difference MRI sequences, according to histological data. Specifically, the EOR was computed on T2-weighted MRI images for Low grade glioma (LGG) and on post-contrast T1-weighted MRI images for High grade glioma (HGG). The median EOR was 94% (range 65%−100%) and 96% (range 50%−100%) in patients who underwent surgery for LGG and HGG, respectively.

### 2.6. Overall Survival

Overall in the study population the 5-year estimated Overall Survival (Oss) was 92%.

### 2.7. Correlation Analysis

We performed a correlation analysis and we found significant negative correlations between both the extent of resection and verbal comprehension (Token test) and between the extent of resection and phonological fluency (r = −371, *p* = 0.02 and r = −36, *p* = −0.027, respectively). In addition, we found significant positive correlations between both histology (LGG; HGG) and working memory and between histology and naming verbs (r = −36, *p* = 0.02 and r = −36, *p* = −0.028, respectively). Sex did not significantly correlate with any measure. Previous oncological treatment was significantly correlated with histology, (r = −0.51, *p* = 0.001).

## 3. Discussion

Despite a modern, multidisciplinary approach in the management of LGG, early relapse and progression will often occur, especially in lesions with a particular molecular pattern [32,33]. In this scenario and given the variability of heterogeneous postoperative treatment for LGG, the role of second surgery outcomes has been poorly investigated [3,16,17,18,25,26,28,29,30,31,34]. The few studies performed on recurrent surgery focus on the neuro-oncological aspect, without considering the neuropsychological point of view, which was the aim of this paper.

Several clinicians are reluctant to re-operate on LGG patients, preferring new cycles of chemotherapy and/or radiotherapy, and avoiding increased surgical risk [27]. Their major concern is related to making further, serious damage to cognitive function in exchange for poor prognostic benefit. More recently, other authors have suggested that surgical resection improves quality of life by reducing the mass effect, incidence of seizures, risk of malignant transformation, and improving overall survival [3,16,17,18,25,26,29,30,31,34]. Fluorescence-guided surgery has also been shown to improve the extent of resection in recurrent gliomas [35]. Despite all these achievements, the authors considered the role of surgery as undefined and that decisions should be made case by case. Their assumption is that there is insufficient evidence to make any specific recommendations [3,16,17,18,25,26,29,30,31,34,35].

Considering the principle that the larger the resection, the longer the survival, which is confirmed by recent literature [1,2,3,4,5,6,7,8,9], this study focuses on the potential change in neuropsychological status of patients who underwent aggressive re-operation. Forty patients have been subject to consecutive homogeneous neuropsychological evaluation, before and after second surgery.

In addition, we analyzed the evolution of neuropsychological changes that might occur after first and second surgery in 17 cases from the same cohort of 40 cases. We followed the entire neurocognitive “path” of these patients, from the first brain surgery until and after the second operation, which has never before been presented. The functional preoperative studies and surgical technique with intraoperative functional mapping used for the first procedure remained the same in the case of the second procedure, considering that the tumor was close to an eloquent area, which was the reason that a tumoral residuum was originally left (see also [36,37]).

Several new studies have shown that the plastic potential of the area may continue with a new functional reorganization that takes place during relapse [19,20,38,39]. This possible behavior of functional areas has allowed the principle of “two-step surgery” in the case of giant lesions.

Moreover, in the cases examined in this study, the main part of the recurrent neoplastic tissue was located either in the previous-tumor cavity or along its walls. This part of the tumor was usually soft, well defined in regard to the parenchyma, and relatively easy to remove. The surgical-functional problem was related to just the infiltrated parenchyma where mapping had been performed (for example, see Figure 2). The only delicate surgical step was the dissection of the scar tissue, which in some cases involved the cortex.

By analyzing the neuropsychological data from 40 patients after second surgery, we considered two different measures: the number of patients scoring within the normal range, namely we tested for changes in the number of patients scoring normally after the second surgery, with respect to the cut-offs, and, as a second measure of the patients’ levels of performance, we tested for changes in their accuracy (number of correct responses).

The analyses were performed by comparing pre- and follow up (4 months after surgery) performances. The patients sample at 4 months included also the 19 patients who had radiotherapy post the second surgery due to an oncological worsening.

As expected, after a short follow up of 4 months, there was no significant difference between patients who underwent radiotherapy and those who did not, but these data just provided us with their status after surgery [40]. These results are reliable since after 4 months the impact of radiotherapy is not likely to be observed.

The neuropsychological tests covered several cognitive domains. Detailed neuropsychological testing was not conducted at terms longer than 4 months because our objective was to analyze the impact of surgery. No significant differences were found for cognitive tests such as naming nouns, comprehension, ideomotor apraxia, short-term memory, working memory, and oral praxis. We found no significant changes either in the number of patients scoring normally between pre- and post-second surgery, or in their accuracy, i.e., their level of performance.

To further analyze the neuropsychological evolution, we considered also the pre- and post- first surgery and post-second surgery data of a smaller sample of 17 patients. We obtained the same results as for the 40 patients used in this study. Only the phonological fluency, considering the level of performance, showed a significant difference in the results between T4 and T1, T2 and T3. The relatively small sample size and the overall good performance limited the results of this correlation analysis between clinical details and neuropsychological data to the results reported just above. The same limitation applies to lesion location. Despite being a small sample size, the set of 17 patients are considered to provide a supplementary study, since the main analyses here involve all 40 patients in the main patient group. Though the sample size used here is relatively small, this study helps to elucidate neuropsychological status in recurrent surgeries, as studies in the literature on this topic are rare.

The neuropsychological status of a patient after a second surgery on a LGG is almost an unknown argument, with only a few studies addressing this aspect. Other studies [21] have reported on the intraoperative mapping of the first and second resection, but there are no previous studies that provide an extensive neuropsychological examination of LGG patients with recurrent surgery. Although our experience and findings are clearly based on a limited number of cases, these preliminary data indicate positive outcomes. These results indicate that second surgery could be considered in cases where surgery with a new extensive resection still remains the better solution for the patient. Further experience and further data are required to validate these findings and draw more generalized conclusions.

## 4. Materials and Methods

### 4.1. Neuropsychological Testing

All participants underwent neuropsychological testing with the same neuropsychologist. The neuropsychological battery was selected to comprehensively cover several cognitive domains, to provide age and years of schooling-corrected normative data, and to provide a short administration time to minimize patient fatigue. For the purpose of the study and statistical analysis we selected specific domains: memory, language (naming nouns, naming verbs, and verbal comprehension) executive abilities (phonological fluency and working memory), and praxis.

The battery of neuropsychological tests involved the assessment of spatio-temporal orientation, handedness (Edinburgh Handedness Inventory [41]), executive functions (working memory [42,43]; verbal fluency [44]), verbal short-term memory (forward [42,43]), praxis (ideomotor limb apraxia [45] and oral apraxia [46]), together with language processing tasks, such as object naming, action naming (battery for the analysis of language disorders [47]), and language comprehension (Token test [48]). The pre-op and post-op assessments in the case of recurrence were indicated T3 and T4, respectively. A subset group of patients (N = 17) were tested at four points in time i.e., before the first surgery (T1), 4 months following the first surgery (T2), one week before recurrence surgery (T3), and 4 months following recurrence surgery (T4).

The local institution ethics committee on human research approved this study, and the patients all signed a consent form.

### 4.2. MRI Structural Data

We retrospectively used T1 and T2 structural imaging data that was routinely acquired during pre-surgery investigations with a 3-T Philips Achieva whole-body scanner.

Structural MRI data processing and statistical analyses were performed using MATLAB r2007b (Mathworks Inc., Natick, MA, USA) and SPM8 (Statistical Parametric Mapping software; Wellcome Department of Imaging Neuroscience, London, UK; http://www.fil.ion.ucl.ac.uk/spm/software/spm8/). Volumes of interest (VOIs) for the patients’ lesions (lesion masks include both the surgical cavity of the first surgery and the lesion regrowth) were drawn on their T2-weighted MRI scans using the MRIcron software (http://www.mccauslandcenter.sc.edu/mricro/mricron/index.html) and were normalized to the Montreal Neurological Institute (MNI) space using the “Clinical Toolbox” (http://www.mccauslandcenter.sc.edu/CRNL/clinical-toolbox) for SPM8.

The MRIcron procedure (http://www.mccauslandcenter.sc.edu/mricro/mricron/index.html) was employed to overlap the lesion templates. An image was generated, selecting the overlap of the lesion masks (VOIs) for the 40 recurring (T3) patient lesions. The number of overlapping lesions is illustrated by different colors that correspond to increasing frequencies (as indicated in the bar code).

### 4.3. Awake Surgery

The decision to re-operate was based primarily on unequivocal evidence of tumor regrowth provided by MRI. The goal of the repeat surgeries remained the maximum resection of the area in the T2-weighted image that was visible on the MRI sequences, including the contrast-enhanced area in the lesions that exhibited anaplastic transformation.

Surgical procedures for first and repeat surgeries were planned using functional MRI and DTI data and conducted under an “awake-online protocol” based on the methodology of Berger and Ojemann [49,50,51] implemented with real-time neuropsychological testing (RTNT), continuous neuropsychological testing during tumor resection enriching the feedback process, as previously described [52].

Patients were fixed to a Mayfield frame and were slightly sedated during exposure and closure of the surgical field [1,53]. Motor evoked potentials (MEPs) and somatosensory evoked potentials (SSEPs) were recorded during surgery to continuously monitor the integrity of motor-sensory pathways (64-channel Eclipse Neurovascular Workstation, Axon Systems Inc.; 32-channel video polygraphic station, Brain Quick SystemPlus, MicroMed). Continuous electrocorticography was performed to detect discharge phenomena and rule out the possibility that the patient’s decrement in performance or oscillatory performance was caused by the concomitant presence of short focal seizures. The EOR was evaluated on post-contrast T1-weighted MRI sequences and on T2-weighted MRI sequences acquired 4 months after surgery for HGG and LGG, respectively [1,54]. For all the tumors in this study, the histological and molecular analysis was conducted and revised according to the World Health Organization (WHO) classification for tumors of the central nervous system [55].

### 4.4. Radiotherapy

Among the patients who exhibited malignant transformation at recurrence, 19 underwent radiation therapy. We considered this variable in the interpretation of the final neuropsychological tests, to determine whether there was any significant difference between patients who had and had not been subject to this treatment.

### 4.5. Statistical Analysis of Neuropsychological Data

The number of patients who scored below the normal range in the consecutive series of 40 patients with recurrent glioma and the subset of 17 patients (i.e., the control group) were analyzed using a McNemar’s test, comparing T3 and T4 in the case of the first series. For the second series we used Cochran’s Q test comparing T1–T4. The neuropsychological scores of the series of 40 patients with recurrent glioma were analyzed using a parametric statistical test (paired t-test) or a non-parametric statistical test (Wilcoxon sign rank-test), depending on whether the variables had a Gaussian distribution. The normal distribution of the data were tested using the Shapiro–Wilk test for normal data. The neuropsychological scores of the subset of 17 patients, who were assessed pre- and post-first and recurrent surgery were analyzed using parametric statistical tests (repeated measures ANOVA). All analyses were conducted using Stata/SE 12.0 for Microsoft Windows. All two-tailed statistical significance levels were set to *p* < 0.05.

## 5. Conclusions

The aim of this study was the evaluation of cognitive aspects in cases of second surgery for tumor resection. Repeat surgery correlates with the oncological assessment that the minor the tumor mass, the better chance of survival follows, although this purely oncological aspect has not been considered here. Our longitudinal follow-up study indicates that glioma surgery is possible without major damage to cognitive functions in the short-term period (4 months) after surgery. Re-operation for recurrent glioma in an eloquent area with accurate neuropsychological assessment and RTNT intraoperative control is an efficacious and safe therapeutic strategy. It is possible to maximize tumor resection while retaining a reduced risk of permanent neurological deficiency, resulting in preservation or even improvement in patient quality of life. This is however a preliminary study with a small number of patients, and further studies with longer follow-up times are necessary to elucidate the conclusive and definitive effects of this surgical approach. The low morbidity associated with re-operation nevertheless suggests that it is preferable to “over indicate” an early re-intervention than to perform a late surgery.

## Figures and Tables

**Table 1:** Patients’ demographic and clinical data (H = hemisphere; P# = case number; M = male; F = female; S = years of schooling; Hist= histology; HGG =high grade glioma; LGG= low grade glioma, EOR = Extent of resection (%), RT = Radiotherapy post-2nd surgery.

P#	Gender	Age	S	Site Surgery	H	Hist	EOR	RT Post 2nd Surgery
1	F	33	13	F-T-I	LH	LGG	66	V
2	M	32	8	F-P	LH	HGG	100	V
3	M	35	13	T-I	LH	HGG	100	V
4	F	52	13	F-P	LH	LGG	85	V
5	M	23	13	F	LH	LGG	100	X
6	M	40	13	T	LH	HGG	100	V
7	F	56	13	F-I	LH	HGG	100	X
8	M	24	13	T-P	LH	HGG	100	X
9	M	36	8	F-T-I	LH	HGG	100	X
10	M	30	18	F	LH	LGG	90	X
11	F	37	18	F-T-I	LH	HGG	85	V
12	M	41	-	F-T-I	LH	HGG	85	V
13	M	20	11	T-P	LH	LGG	65	X
14	M	49	17	F-T-I	LH	HGG	90	V
15	M	45	18	F	LH	LGG	90	X
16	M	44	13	T	LH	HGG	100	V
17	M	35	8	F-P	LH	HGG	100	V
18	F	34	8	F-P	LH	HGG	100	X
19	F	38	18	F-P	LH	LGG	85	X
20	M	48	8	F-T-I	LH	HGG	100	V
21	M	37	13	F-T-I	LH	LGG	90	X
22	F	17	10	F	LH	LGG	85	X
23	M	39	8	F	LH	HGG	100	V
24	F	66	5	F-T-I	LH	HGG	100	V
25	M	45	18	F-T-I	LH	LGG	70	X
26	M	47	18	F-T-I	LH	HGG	100	V
27	M	57	18	T	LH	HGG		X
28	M	15	10	F-I	LH	LGG	100	X
29	M	29	18	F-I	LH	LGG	100	X
30	M	32	5	F	LH	HGG	84	X
31	M	36	5	F	LH	HGG	100	V
32	F	48	13	F-T-I	LH	HGG	50	V
33	M	33	8	F-I	LH	LGG	80	V
34	M	36	8	F-I	LH	HGG	85	X
35	M	34	18	F-T-I	LH	LGG	85	X
36	F	28	13	F-T-I	LH	HGG	100	V
37	M	18	12	T	LH	LGG	100	X
38	F	34	13	F-I	LH	LGG	92	X
39	F	35	13	F-I	LH	LGG	90	X
40	F	47	8	T	LH	HGG	100	V

**Table 2:** Number of patients with recurrent glioma whose results were within the normal range at T3 and T4 (pre and post-recurrence surgery, respectively), and the results of the McNemar test (T3 vs. T4). We stratified patients who worsened (w, those within the normal range at T3 who were below the normal range at T4), and improved (i, those below the normal range at T3 who were within the normal range at T4). The mean and standard deviation of patients who scored below the normal range (and the cut-off) are shown in grey. To date, the level of performance of those patients was only slightly below the cut-offs.

Number of Patients within the Normal Range				Level of Performance of Patients Below the Normal Range		
Test	T3	T4	T3 vs. T4 McNemar	T3	T4	Cut-offs
STM	31/38	28/38 (4/38 w; 1/38 i)	*p* = 0.375, n.s.	3.05 ± 1.4	3.39 ± 0.41	4.26
o_PRAXIS	40/40	40/40	-	-	-	-
IMA	38/38	38/38	-	-	-	-
Compr	35/39	35/39 (1/39 w, 1/39 i)	*p* = 1, n.s.	22.75 ± 1.55	23.63 ± 1.80	26.26
N_nam	30/40	27/40 (4/40 w, 1/40 i)	*p* = 0.37, n.s.	25.60 ± 1.78	25.23 ± 2.05	28
v_nam	24/39	25/39 (4/39 w, 5/39 i)	*p* = 1, n.s.	23.13 ± 1.85	23.57 ± 1.45	26
WM	25/31	23/31 (4/35w, 2/31 i)	*p* = 0.68, n.s.	2.32 ± 0.31	2.26 ± 0.39	2.65
Ph_Fl	32/38	26/38 (7/38 w, 1/38 i)	*p* = 0.07, n.s.	11 ± 5.19	8 ± 5.33	16

STM = short-term memory, o_PRAXIS = oral praxis, IMA = ideomotor apraxia, Compr = verbal comprehension, N_nam = noun naming, v_nam = verb naming, WM = working memory, Ph_Fl = phonological fluency.

**Table 3:** Shapiro–Wilk test, using a = 0.05, on the normality distribution of the patients’ level of performance.

Task	Pre-Surgery	Post-Surgery
WM	*p* = 0.28	*p* = 0.74
Ph_Fl	*p* = 0.19	*p* = 0.51
Compr	*p* < 0.001	*p* < 0.001
o_PRAXIS	*p* < 0.001	*p* < 0.001
STM	*p* = 0.01	*p* = 0.2
IMA	*p* < 0.001	*p* < 0.001
N_nam	*p* < 0.001	*p* < 0.001
v_nam	*p* < 0.001	*p* < 0.01

STM = short-term memory, o_PRAXIS = oral praxis, IMA = ideomotor apraxia, Compr = verbal comprehension, N_nam = noun naming, v_nam = verb naming, WM = working memory, Ph_Fl = phonological fluency.

**Table 4:** Results of the student *t*-test or Wilcoxon test comparing pre-surgery (T3) to post-surgery (T4) patients’ level of performance.

Task	T3	T4	T3 vs. T4
STM	5.04 ± 1.23	5 ± 1.18	Z = −0.5, *p* = 0.59
WM	3.63 ± 1.13	3.63 ± 1.14	t (30) = −0.18 *p* = 0.5
IMA	71.38 ± 1.44	70.55 ± 3.28	Z = 0.84, *p* = 0.39
N_nam	28.23 ± 1.9	27.95 ± 2.35	Z = 0.416, *p* = 0.67
v_nam	25.79 ± 1.9	25.92 ± 2.08	Z = −0.506, *p* = 0.612
Compr	31.37 ± 3.5	31.49 ± 3.22	Z = −0.379, *p* = 0.750
Ph_Fl	27.38 ± 12.29	25.26 ± 15.07	t (37) = 0.907, *p* = 0.18
o_PRAXIS	19.5 ± 1.19	19.30 ± 1.58	Z = −2.44, *p* = 0.014

STM = short-term memory, o_PRAXIS = oral praxis, IMA = ideomotor apraxia, Compr = verbal comprehension, N_nam = noun naming, v_nam = verb naming, WM = working memory, Ph_Fl = phonological fluency.

**Table 5:** Number of the 17 patients with recurrent glioma whose results were within the normal range at time points T1 and T2 (pre- and post-first surgery, respectively) and at T3 and T4 (pre- and post-recurrence surgery, respectively), with Cochran’s Q test of equality of proportions between the different times points. We stratified patients who decreased (w, normal range at T3 and below the normal range at T4), and improved (i, below the normal range at T3 but within the normal range at T4). The mean and s.d. below the normal patient range for patient performance (and the cut-off) are shown in grey. To date, the level of performance in those patients was only slightly below the cut-offs (reported in the last column).

Task	Number of Patients within the Normal Range					Level of Performance of Patients Below the Normal Range				
	T1	T2	T3	T4	T1–T4 Cochran’s Q test	T1	T2	T3	T4	Cut-offs
STM	10/13	12/13 (0 w,2 i)	11/13	10/13 (1w, 0i)	*p* = 0.299, n.s.	3.56 ± 0.15	3.65 ± 0.84	3.58 ± 0.26	3.38 ± 0.45	4.26
o_PRAXIS	16/16	16/16	16/16	16/16	-	-	-	-	-	16
IMA	17/17	17/17	17/17	17/17	-	-	-	-	-	52
Compr	17/17	17/17	17/17	17/17	*p* = 0.391, n.s.	-	-	22	26	26.26
N_nam	12/17	14/17 (0w, 2 i)	13/17	12/17 (1w, 0i)	*p* = 0.468, n.s.	25.67 ± 1.34	25.67 ± 0.58	24.50 ± 2.38	23.80 ± 2.77	28
v_nam	8/12	8/12 (2w, 2 i)	7/12	9/12 (1w, 3 i)	*p* = 0.801, n.s	22.25 ± 1.71	24 ± 2.5	23.43 ± 2.37	23 ± 1.63	26
WM	6/8	6/8 (0w, o i)	7/8	6/8 (1w, 0 i)	*p* = 0.732, n.s.	2.28 ± 0.99	2.16 ± 0.99	2.28 ± 1.70	1.70 ± 0.37	2.65
Ph_Fl	9/13	12/13 (o w, 3 i)	11/13	8/13 (3w, 0i)	*p* = 0.057, n.s.	10 ± 7.35	14 ± 2.83	10 ± 7.94	2.60 ± 1.95	16

**Table 6:** Repeated measures ANOVA results for patients’ level of performance in various tasks pre- and post-first surgery (T1 and T2, respectively) and pre- and post-recurrence surgery (T3 and T4, respectively).

Task	Cut-offs	T1	T2	T3	T4	Repeated Measures ANOVA	Post-Hoc Analyses (Tukey-Corrections)
Ph_Fl	16	28.92 ± 18.16	31.9 ± 15.75	30.24 ± 14.69	25.06 ± 17.94	F(3.36) = 8.18, *p* = 0.0003	T4 vs. T1. t(12) = −3.09, *p* = 0.019 T4 vs. T2 t(12) = −4.66, *p* < 0.001 T4 vs. T3 t(12) = −3.75, *p* = 0.003
N_nam	28	28.71 ± 2.17	28.59 ± 1.66	28.06 ± 2.41	27.76 ± 3.07	F(3.48) = 1.13, *p* = 0.33	-
v_nam	26	26.00 ± 2.92	26.36 ± 2.27	25.88 ± 2.62	26.35 ± 2.15	F(3.33) = 0.42, *p* = 0.67	-
Compr	26.26	33.01 ± 1.15	32.84 ± 1.09	32.00 ± 2.81	31.94 ± 1.94	F(3.48) = 3.06, *p* = 0.07	-
IMA	52	70.82 ± 3.00	71.59 ± 1.00	71.53 ± 1.07	71.40 ± 1.59	F(3.42) = 0.67, *p* = 0.48	-
STM	4.26	4.91 ± 1.36	4.81 ± 0.72	5.14 ± 0.99	5.08 ± 1.23	F(3.36) = 0.34, *p* = 0.78	-
WM	2.65	3.61 ± 1.37	3.80 ± 1.36	3.75 ± 1.38	3.75 ± 1.22	F(3.21) = 0.69, *p* = 0.568	-
o_PRAXIS	26	19.69 ± 0.25	19.75 ± 0.00	19.34 ± 1.70	19.28 ± 1.94	F(3.45) = 1, *p* = 0.33	-

STM = short-term memory, o_PRAXIS = oral praxis, IMA = ideomotor apraxia, Compr = verbal comprehension, N_nam = noun naming, v_nam = verb naming, WM = working memory, Ph_Fl = phonological fluency.

**Table 7:** Effect of performing radiotherapy post-recurrent surgery: comparison of performance between patients who did and did not undergo radiotherapy.

Task		Level of Performance	
Test	T-Test	RT	No RT
STM	t(37) = 0.32, *p* = 0.74, n.s.	5.06 ± 1.10	4.93 ± 1.28
o_PRAXIS	t(38) = 0.116, *p* = 0.908, n.s.	19.32 ± 1.43	19.27 ± 1.74
IMA	t(36) = −0.98, *p* = 0.33, n.s.	70 ± 3.99	
Compr	t(38) = −0.53, *p* = 0.59, n.s.	31.20 ± 3.42	31.75 ± 3.11
N_nam	t(38) = −0.67, *p* = 0.504, n.s.	27.68 ± 2,75	28.19 ± 1.97
v_nam	t(37) = −1.02, *p* = 0.314, n.s.	25.56 ± 2,09	26.24 ± 2.07
WM	t(33) = 0.704, *p* = 0.48, n.s.	3.78 ± 1.28	3.504 ± 1.04
Ph_Fl	t(37) = 0.05, *p* = 0.96, n.s.	25.39 ± 13.41	25.14 ± 16.70

STM = short-term memory, o_PRAXIS = oral praxis, IMA = ideomotor apraxia, Compr = verbal comprehension, N_nam = noun naming, v_nam = verb naming, WM = working memory, Ph_Fl = phonological fluency.
